# Self-Supervised Learning with Trilateral Redundancy Reduction for Urban Functional Zone Identification Using Street-View Imagery

**DOI:** 10.3390/s25051504

**Published:** 2025-02-28

**Authors:** Kun Zhao, Juan Li, Shuai Xie, Lijian Zhou, Wenbin He, Xiaolin Chen

**Affiliations:** School of Information and Control Engineering, Qingdao University of Technology, Qingdao 266520, China; zhaokun@qut.edu.cn (K.Z.); jr78one@gmail.com (J.L.); xieshuai@radi.ac.cn (S.X.); zhoulijian@qut.edu.cn (L.Z.); binbin12019@outlook.com (W.H.)

**Keywords:** street-view imagery, self-supervised learning, redundancy reduction, urban scene classification, urban functional zone identification

## Abstract

In recent years, the use of street-view images for urban analysis has received much attention. Despite the abundance of raw data, existing supervised learning methods heavily rely on large-scale and high-quality labels. Faced with the challenge of label scarcity in urban scene classification tasks, an innovative self-supervised learning framework, Trilateral Redundancy Reduction (Tri-ReD) is proposed. In this framework, a more restrictive loss, “trilateral loss”, is proposed. By compelling the embedding of positive samples to be highly correlated, it guides the pre-trained model to learn more essential representations without semantic labels. Furthermore, a novel data augmentation strategy, tri-branch mutually exclusive augmentation (Tri-MExA), is proposed. Its aim is to reduce the uncertainties introduced by traditional random augmentation methods. As a model pre-training method, Tri-ReD framework is architecture-agnostic, performing effectively on both CNNs and ViTs, which makes it adaptable for a wide variety of downstream tasks. In this paper, 116,491 unlabeled street-view images were used to pre-train models by Tri-ReD to obtain the general representation of urban scenes at the ground level. These pre-trained models were then fine-tuned using supervised data with semantic labels (17,600 images from BIC_GSV and 12,871 from BEAUTY) for the final classification task. Experimental results demonstrate that the proposed self-supervised pre-training method outperformed the direct supervised learning approaches for urban functional zone identification by 19% on average. It also surpassed the performance of models pre-trained on ImageNet by around 11%, achieving state-of-the-art (SOTA) results in self-supervised pre-training.

## 1. Introduction

Street-view imagery is interactive panoramic representation. It provides geographic information about locations along roadways. It is akin to a lateral view of urban landscapes, captured from the perspective of human vision [1]. Compared to remote sensing (RS) images, street-view images (SVIs) can capture finer-grained, detailed information, making it a crucial data source for urban functional zone identification [2]. With the widespread popularity of electronic maps such as Google Maps, SVIs have emerged as a focal point of research due to the advantages conferred by low viewing angles, the ease of accessibility, and other characteristics that facilitate richer visual information [3]. Therefore, SVIs are increasingly being utilized in a variety of fields, including urban planning and research [4], the assessment of urban renewal potential [5], autonomous driving [6], and smart cities [7].

As the volume of urban street-view image data rapidly expands, a crucial issue emerges. Compared to the vast amount of data, there are a lack of corresponding semantic annotations. The manpower and resources required for large-scale and high-quality annotations are considerable [8]. While some open crowdsourced geographic information platforms (e.g., OpenStreetMap, OSM) have been employed to alleviate this issue by assigning semantic labels to corresponding SVIs (e.g., Google Street View) [9] through geographical location matching, disparities in coordinate systems among different systems and the inconsistent quality of crowdsourced annotation still result in a scarcity of high-quality annotations. For example, ref. [9] provides only approximately 20,000 sample annotations for nearly 140,000 SVIs. The existence of this problem presents a formidable obstacle to effectively classifying these massive amounts of street images. When confronted with label scarcity in supervised learning, self-supervised Learning (SSL) methods [10,11,12,13,14,15,16,17] demonstrate immense potential. SSL autonomously introduces latent supervisory signals by treating each sample and its variants as separate classes. Through learning from these “self-supervised signals”, SSL effectively explores and represents the internal structure of massive data without semantic annotations. However, due to the absence of task-specific guidance during training, SSL often requires the application of various special tricks in order to avoid model collapse. For instance, contrastive learning [10,11,12,13] relies on large-scale negative sample pairs to constrain the training process, while predictive learning [14,15,16] achieves model simplification by not requiring negative sample pairs but relying on complex training techniques such as self distillation [14,16] and alternating stop-gradient [15], etc. In recent years, SSL has been used in the field of remote sensing [18,19,20,21,22,23,24], where pre-training methods typically incorporate the aforementioned techniques. However, there is limited research on SSL for SVIs. As a special type of scene imagery with limited visual elements but diverse arrangements and forms of elements, urban street-view imagery contains both fine-grained visual features and high-level abstract semantics simultaneously [25]. This presents a greater challenge for model pre-training using SSL.

**Figure 1:**
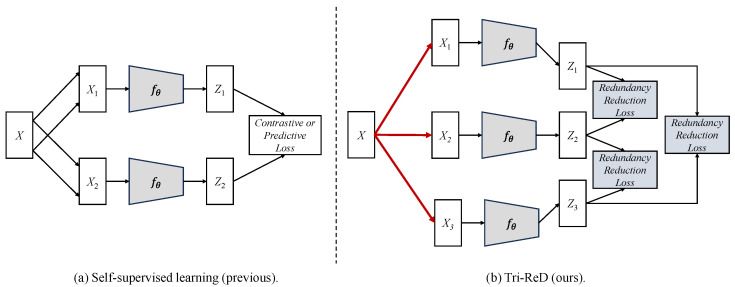
Concept map of self-supervised learning methods: (**a**) previous SSL and (**b**) our proposed Tri-ReD. The red lines highlight the innovative data augmentation strategy presented in this paper.

Inspired by Barlow Twins [17] and InfoMin [26], the Trilateral Redundancy Reduction (Tri-ReD) framework is proposed in this paper to enhance the representational capacity and training stability of SSL methods for urban street scene image analysis. Instead of seeking to maximize mutual information, Tri-ReD simplifies the training process by aiming for “just enough” information. Tri-ReD eliminates the need for negative samples and complex self-distillation strategies to enhance training stability. It reduces training complexity and simultaneously enhances model stability by introducing redundancy reduction. More specifically, Tri-ReD employs a trilateral pairwise mapping strategy which naturally increases the differences between positive samples, thereby preventing model collapse and allowing it to focus more on consistency learning between positive samples. The key distinctions between Tri-ReD and existing SSL methods are shown in Figure 1.

By introducing self-supervised learning, an innovative model pre-training solution is provided in this paper to address the lack of high-quality annotations in urban street scene imagery. The main contributions of this study are as follows.

- Trilateral Redundancy Reduction (Tri-ReD) Framework: For street scene image data with fine-grained visual distinctions, traditional dual-path loss in SSL often falls short in representation capability. To ensure that the model captures more comprehensive information, we propose the Tri-ReD framework, which facilitates the learning of invariant features and simultaneously stabilizes the training process.
- Tri-branch Mutually Exclusive Augmentation (Tri-MExA) Strategy: We also propose a Tri-MExA strategy based on the Tri-ReD framework. This strategy can increase the diversity and randomness of data. It does so without relying on excessive strong augmentation techniques, thereby contributing to more stable model training.
- Simulated Vegetation Color Occlusion (SVCO) Data Augmentation: We designed a novel data augmentation method, SVCO. It serves as the third branch, specifically tailored for the SSL of SVIs. This method enhances the model’s suitability for different downstream tasks and avoids excessive differences in data distribution that result in learning irrelevant feature representations.

The remainder of this paper is organized as follows. Section 2 reviews the related work involved in this paper. Section 3 introduces the related datasets, primary methods proposed, and experimental settings in this paper. Section 4 shows quantitative and qualitative analysis of Tri-ReD. Section 5 further discusses the restriction and redundancy reduction of Tri-ReD. Finally, Section 6 concludes this paper.

## 2. Related Work

The development of supervised learning methods in remote sensing has been impeded by the lack of large-scale labeled datasets comparable to ImageNet [27]. Recently, SSL has achieved favorable outcomes on various downstream tasks such as image classification, object detection, and semantic segmentation, even surpassing the performance of supervised pre-trained models [11]. In this section, we briefly review the relevant studies on urban functional zone identification based on SVIs, as well as the latest advancements in SSL methods used in remote sensing tasks.

### 2.1. Scene Classification for Urban Functional Zone Identification Using Street-View Images

Image classification is one of the most crucial research tasks in computer vision (CV). Its goal is to assign appropriate labels to given images based on their content, typically corresponding to different semantic categories. SVIs provide abundant ground-level information, offering opportunities for exploring land use and land cover (LULC). In urban scene classification, most studies focus on feature extraction, model design, or data augmentation. Manual feature engineering in traditional CV is utilized to characterize image scenes. For example, in [28], features including the GIST, Histogram of Oriented Gradient (HoG), and Scale-Invariant Feature Transform-Fisher (SIFT-Fisher) were extracted from SVIs. Nevertheless, such low-level representations are inadequate and inefficient.

The advancement of deep learning (DL) and CV has made it possible to automatically and efficiently extract high-level semantic information from image data. The application of SVIs in various fields has primarily benefited from these advancements. A series of innovative DL algorithms has emerged [29,30,31,32,33], laying a solid foundation for urban street-view image analysis. Ref. [34] employed convolutional neural networks (CNNs) to develop an indoor/outdoor classifier for ground-level images, which were then used to identify eight parcel-based land use categories within Stanford University’s campus. As supplementary to remote sensing data, SVIs were used to map land use at the parcel level in a megacity [35]. Urban buildings were classified into eight categorizes by using detailed building function information from OSM to mark buildings in SVIs [9]. The types of buildings and their materials were automatically detected by employing CNNs [36], which provided essential references for enhancing seismic risk assessment and urban planning. An ensemble learning method was proposed by [37] to address urban land classification tasks involving multiple data sources (e.g., RS, SVIs). Ref. [25] designed a “Detector–Encoder–Classifier” framework, which acquires building bounding boxes from the detector and subsequently encodes contextual information to partition urban functional zones. Additionally, Ref. [38] introduced a multimodal strategy that integrates visual and language modalities for fine-grained land use classification, using SVIs and spatial context-aware land use descriptions.

While SVIs have been extensively implemented in LULC tasks, the majority of studies have focused on the supervised learning paradigm, which requires costly data annotation. Whereas unlabeled data are relatively easy to obtain, there have been limited studies on using unlabeled street-view image data for related tasks. This highlights the growing importance of combining SSL methods with SVIs.

### 2.2. Self-Supervised Learning

Self-supervised learning is a form of unsupervised learning that leverages a large amount of unlabeled data to learn representations beneficial for downstream tasks. Although the concept of self-supervised learning is not entirely novel, having originated in robotics and later adopted by the machine learning community [8], it remains a highly active and promising topic. SSL is recognized as a key element in the future of artificial intelligence (AI). SSL has shown great potential in various fields such as natural language processing (NLP) and computer vision [39]. Early research in SSL focused on learning feature representations from abundant unlabeled data. For instance, autoencoders effectively learn representations by reconstructing input data [40], while Word2vec captures semantic information by generating vector representations of words [41,42]. SSL has been popularized by major advancements in deep learning, especially in recent years. In 2018, the BERT model [43] made a substantial impact by achieving SOTA performance by employing a masked language model and next-sentence prediction. Nevertheless, one limitation of this method is the lack of input masked tokens that can be utilized for downstream tasks.

SSL has nearly rivaled or even surpassed the performance of supervised learning in many downstream tasks. Contrastive learning is one of the dominant components in SSL, which has demonstrated remarkable performance by learning feature representations through a comparative approach. MoCo [11] and SimCLR [12] are two typical contrastive learning methods that use negative samples to help feature representation learning. Specifically, MoCo used a queue to manage negative samples and employed a momentum-based approach to update the encoder, while SimCLR emphasized the critical role of combining data augmentation, introducing a novel framework that does not rely on momentum or memory bank. Despite these methods’ significant achievements, they still have some drawbacks such as the complexity of training and the difficulty in negative sample selection. In contrast, BYOL [14], Barlow Twins [17], and DINO [16] indicate that negative samples are unnecessary. BYOL learns image representations by predicting a previous version of its outputs, relying on two neural networks. In detail, the target network is updated based on the online network by using an exponential moving average approach. BYOL also relies on existing augmentation sets specific to visual applications. However, since vision datasets can be biased, the learned representations might not always be broadly applicable. The training of SSL was further simplified by LeCun team. The predictive task was adjusted to harmonize the consistency of features among positive sample pairs and decouple feature elements. A method of knowledge distillation without labels was proposed by DINO, in which a student network is trained to mimic a more powerful teacher network. Global self-attention mechanisms are used to capture more comprehensive views of data, rather than relying on negative samples.

A large number of related works have also emerged in the field of remote sensing. Based on contrastive learning, remote sensing images captured from the same location but by different satellite sensors are considered positive pairs, while those from different locations and sensors are considered negatives. Ref. [21] validated the practicality of transformer models in earth observation tasks. A prototype contrastive learning framework [24] was proposed to address cross-domain classification tasks in remote sensing. In [44], ground-level panoramic images were initially mapped to bird’s-eye-view images to generate pseudo-satellite images. The model, trained based on contrastive learning, then learned cross-view feature representations. Finally, the location of ground images was inferred by comparing the similarity between the ground-view and satellite images. A dual-stream contrastive learning network [45] was designed for hyperspectral image classification. It is dedicated to exploring both spatial and spectral diversity among samples. In our work, we propose a trilateral pairwise mapping strategy and a mutually exclusive data augmentation strategy to enhance performance and ensure greater stability during the training process.

## 3. Materials and Methodology

**Figure 2:**
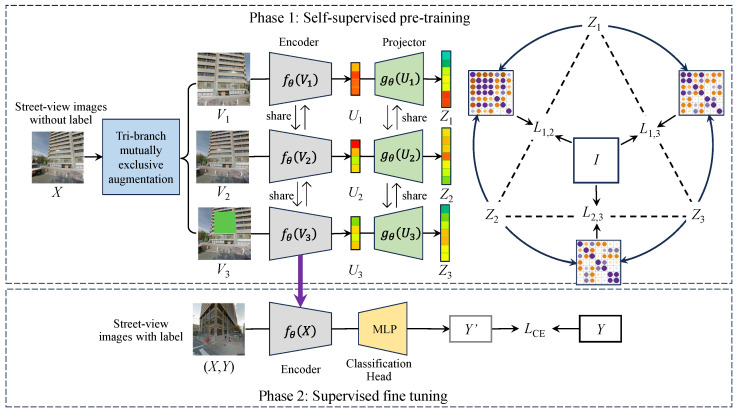
The overall framework diagram of the proposed Tri-ReD, where *I* is the identity matrix and $M_{i,j}$ is the cross-correlation matrix of $Z_{i}$ and $Z_{j}$.

In this section, we introduce the datasets, proposed method, and experimental design. Figure 2 shows the pipeline of the proposed approach. In Phase 1, a large collection of unlabeled SVIs are utilized to pre-train the model through self-supervised learning. During this process, the features representations are learned by the model. In Phase 2, the pre-trained backbone model from Phase 1 is transferred to a specific downstream task, such as image classification. Since the last fully connected (FC) layer of the encoder is replaced with an identity layer in Phase 1, the identity layer is replaced with a classification head that matches the number of categories in the dataset in Phase 2. In this process, the model is fine-tuned using a small amount of labeled SVIs to improve image classification performance under the condition of limited labeled data. To verify the effectiveness of the proposed method, a series of ablation experiments based on the components of Tri-ReD, as well as comparative experiments between the proposed method and existing SSL methods, were designed. The experiments were conducted on the public BIC_GSV [9] dataset for urban ground object classification and the public BEAUTY [25] dataset for urban functional zone identification, respectively. This evaluation aimed to verify the advancement of the proposed method in urban scene classification tasks at different levels.

### 3.1. Dataset Description

The dataset is divided into two subsets: the unlabeled dataset and the labeled dataset. The unlabeled dataset, employed for self-supervised pre-training, is characterized by its absence of label information and substantial data volume. Conversely, the labeled dataset is smaller but highly accurate. It is painstakingly labeled by human experts and designated for various downstream tasks. A detailed description of the dataset is given below.

#### 3.1.1. Unlabeled Dataset

**Figure 3:**
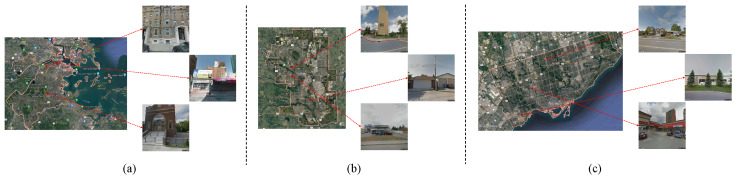
Presentation of CSM dataset samples: (**a**) collected in Boston; (**b**) collected in Calgary; (**c**) collected in Toronto.

- City-Scale Maps (CSM (CSM & BIC_GSV: https://syncandshare.lrz.de/dl/fiTFS5He9bZsR4Urh8hZGDGg/BIC_GSV.tar.gz (accessed on 25 February 2025))) [9]: The CSM dataset is comprised of 116,491 samples collected from major cities in the United States and Canada, including Boston, Calgary, and Toronto. The specific geographical locations of three samples from each of these cities are presented in Figure 3.

#### 3.1.2. Labeled Dataset

**Figure 4:**
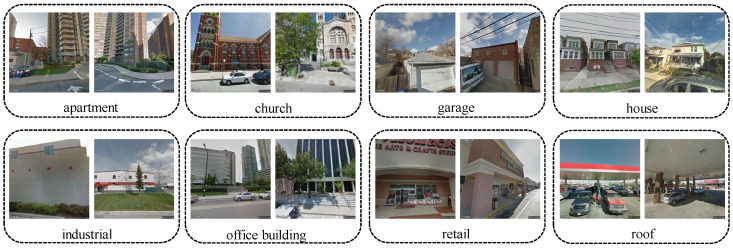
Example images of BIC_GSV dataset.

**Figure 5:**
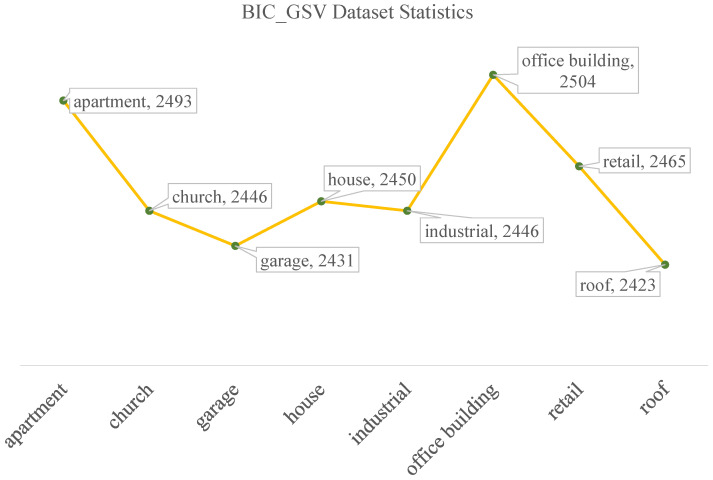
Statistics of BIC_GSV dataset.

**Figure 6:**
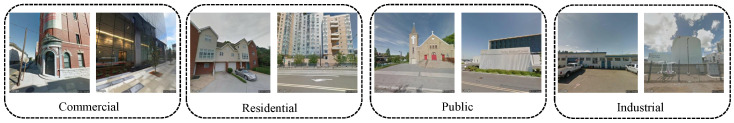
Example images of BEAUTY dataset.

**Figure 7:**
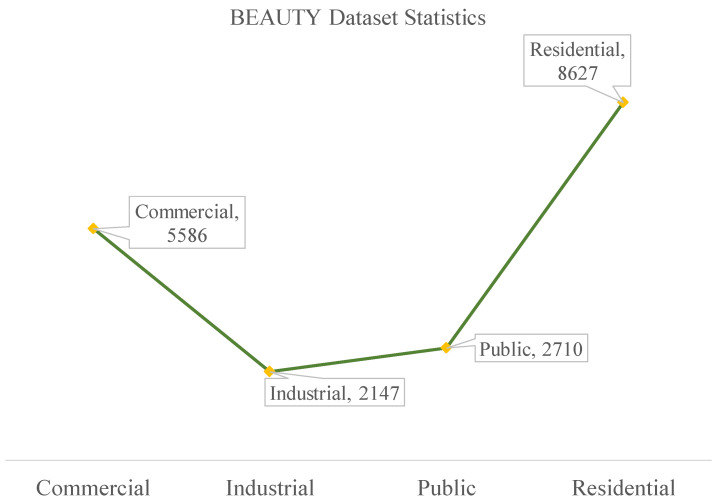
The statistics of BEAUTY dataset.

- BIC_GSV: There are a total of 19,658 samples in the BIC_GSV dataset, which is categorized into eight urban ground object classes: apartment, church, garage, house, industrial, office building, retail, and roof. Sample images and detailed information are shown in Figure 4 and Figure 5, respectively.
- BEAUTY (BEAUTY: https://pan.baidu.com/share/init?surl=nSPU68H36CMsn5pboD5e9A&pwd=T78D (accessed on 25 February 2025)): The BEAUTY dataset consists of 19,070 samples in total. It is divided into four abstract functional zones, namely commercial, residential, public, and industrial. Examples from the BEAUTY dataset are shown visually in Figure 6, with detailed information provided in Figure 7.

### 3.2. Method Description

#### 3.2.1. Overview

Given that $X\in R^{H\times W\times C}$, where *H*, *W*, and *C* represent the height, width, and number of channels, respectively, then each input sample *X* in the mini-batch is augmented:

(1) $$V_{i}=\Gamma_{i}\left(X_{i}\right),$$

where *V* represents the augmented image and Γ denotes the data augmentation method.

Then, each augmented image is passed on to an encoder, yielding vector representations:

(2) $$U_{i}=f_{\theta }\left(V_{i}\right),$$

where *U* represents the intermediate embedding obtained from the encoder $f_{\theta }$.

Finally, the vector representations are used to calculate the loss:

(3) $$Z_{i}=g_{\theta }\left(U_{i}\right),$$

where *Z* represents the feature representation obtained through the projector $g_{\theta }$, which is utilized for computing the loss.

As shown in Figure 2, during the model pre-training process (Phase 1), our proposed Tri-ReD is composed of three parallel paths, each containing an encoder and a projector. The encoder can be built on any CNN or vision transformer backbone. In our case, we used ResNet18 as the baseline encoder. The projector is implemented as a multi-layer perceptron (MLP). Firstly, Tri-ReD applies tri-branch mutually exclusive data augmentations to the input images. Next, the features are extracted from these augmented images by the encoder and the projector is used for dimensional expansion. Subsequently, the resulting feature representations are mapped pairwise to capture the different information carried by various feature vectors, ensuring that the effective information can be fully exploited.

#### 3.2.2. Tri-Branch Mutually Exclusive Augmentation

The objective of data augmentation is to increase the variability of the data and expose the model to different perspectives of the same instance. In the field of image classification, data augmentation plays a crucial role in enhancing the robustness and generalization capability of models. Selecting appropriate data augmentation methods is a key step in training deep learning models. To fully exploit the inherent information within the dataset, a Tri-MExA strategy based on the characteristics of the dataset is proposed in this paper. Augmentations are carefully designed to preserve the effective distribution of data and improve the performance of the model. Firstly, when using a dual-path random data augmentation strategy, there is a probability that identical data samples will be generated, which may hinder the model’s ability to extract meaningful information from these similar samples. Secondly, overusing augmentations may lead to excessive distortions, where the variance along the feature directions caused by data augmentation may exceed that of the data distribution, potentially resulting in model collapse [46]. Thirdly, redundancy is likely to be introduced by similar data augmentation strategies. As a result, the challenge of redundancy reduction and the difficulty of model training are significantly increased. Moreover, noise could be introduced by inappropriate data augmentation, which significantly deviates from the real data. When the disparity between the data distribution of the downstream task and the augmented data is substantial, irrelevant or detrimental features might be learned by the model. Taking the example of an SVI, the sky typically occupies the upper portion of the image, while the ground is at the bottom. The application of random vertical flipping misaligns with these real-world characteristics of the SVI. Therefore, the model’s ability to capture useful features from real data is probably diminished.

In order to maximize the extraction of feature information from the data, we deliberately designed a tri-branch mutually exclusive augmentation strategy. It is tailored to the specific characteristics of the dataset, rather than simply relying on stacking numerous data augmentations. This strategy is grounded in a profound understanding of the data, taking into account its authentic features. By implementing Tri-MExA, the model can learn a more diverse set of image views, allowing the information across different views to complement other information. Consequently, more reliable and meaningful training samples are provided for the model and the comprehension of the essence of the data is enhanced.

**Figure 8:**
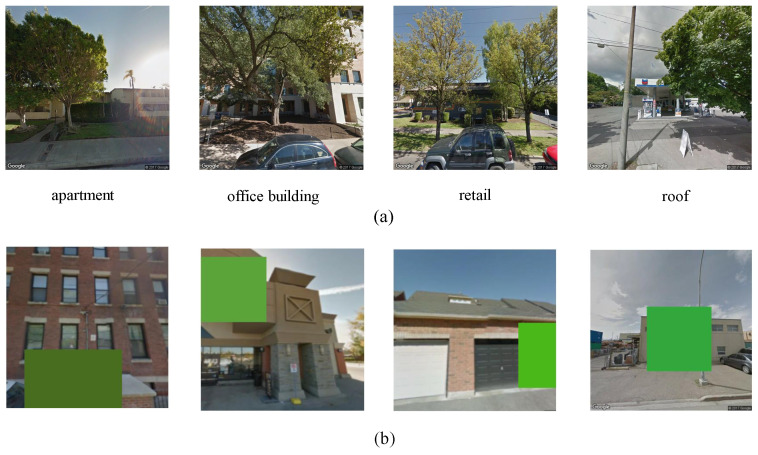
Samples of (**a**) severe occlusion in dataset and (**b**) simulated vegetation color occlusion.

Specifically, we apply distinct data augmentations to each branch. Apart from traditional augmentation methods, we propose a novel data augmentation method that simulates vegetation color occlusion (SVCO). Each branch of Tri-MExA involves Horizontal Flip, Color Jitter, and SVCO, respectively. Additionally, each branch undergoes preprocessing steps such as Resize, Center Crop, and Random Crop. Our proposed SVCO is shown in Figure 8.

As depicted in Figure 8a, buildings are hidden by trees in the immediate vicinity. In response, a data augmentation method, SVCO, was devised (see Figure 8b). It is based on a comprehensive analysis and understanding of the dataset’s attributes. Since the dataset mainly consists of street-level images, severe occlusion issues are primarily caused by trees. Therefore, SVCO is included as one of the branches in Tri-MExA to better emulate common visual obstacles encountered in real world scenarios.

Let *X* denote the original image and *M* represent the mask for vegetation regions, with *M* and *X* having consistent dimensions. The operation of simulating occlusion with vegetation color can be expressed using the following formula:

(4) Y=X⊙(1−M)+color_trees⊙M,

where *Y* denotes the image after applying SVCO, ⊙ denotes element-wise multiplication, and color_trees represents the color simulated to mimic vegetation.

SVCO not only enhances the model’s capability to discern occluded scenarios but also facilitates the learning of adaptive feature representations for tackling occlusion. Moreover, a more diverse set of challenging training samples related to occlusion are introduced by this data augmentation method. These contributions significantly improve the model’s generalization and robustness.

#### 3.2.3. Trilateral Redundancy Reduction

Different features may carry distinct information. In order to better explore feature correlations and precisely measure losses, Trilateral Redundancy Reduction (Tri-ReD) loss is designed. This loss function maps pairwise features to separate cross-correlation matrices, allowing for “just enough” mutual information. This restriction helps the model capture the intricate relationships between these features more effectively. As a result, the effectiveness of feature representation is enhanced, and a novel perspective for exploring the intrinsic connections between image features is offered. In our paper, we quantify the correlation between features as follows:

(5) $$L_{1,2}=\sum_{i}{\left(I-\frac{\sum_{b}Z_{b,i}^{1}Z_{b,i}^{2}}{\sqrt{\sum_{b}{\left(Z_{b,i}^{1}\right)}^{2}}\sqrt{\sum_{b}{\left(Z_{b,i}^{2}\right)}^{2}}}\right)}^{2}+\alpha_{1}\sum_{i}\sum_{j\neq i}{\left(\frac{\sum_{b}Z_{b,i}^{1}Z_{b,j}^{2}}{\sqrt{\sum_{b}{\left(Z_{b,i}^{1}\right)}^{2}}\sqrt{\sum_{b}{\left(Z_{b,j}^{2}\right)}^{2}}}\right)}^{2},$$

(6) $$L_{1,3}=\sum_{i}{\left(I-\frac{\sum_{b}Z_{b,i}^{1}Z_{b,i}^{3}}{\sqrt{\sum_{b}{\left(Z_{b,i}^{1}\right)}^{2}}\sqrt{\sum_{b}{\left(Z_{b,i}^{3}\right)}^{2}}}\right)}^{2}+\alpha_{2}\sum_{i}\sum_{j\neq i}{\left(\frac{\sum_{b}Z_{b,i}^{1}Z_{b,j}^{3}}{\sqrt{\sum_{b}{\left(Z_{b,i}^{1}\right)}^{2}}\sqrt{\sum_{b}{\left(Z_{b,j}^{3}\right)}^{2}}}\right)}^{2},$$

(7) $$L_{2,3}=\sum_{i}{\left(I-\frac{\sum_{b}Z_{b,i}^{2}Z_{b,i}^{3}}{\sqrt{\sum_{b}{\left(Z_{b,i}^{2}\right)}^{2}}\sqrt{\sum_{b}{\left(Z_{b,i}^{3}\right)}^{2}}}\right)}^{2}+\alpha_{3}\sum_{i}\sum_{j\neq i}{\left(\frac{\sum_{b}Z_{b,i}^{2}Z_{b,j}^{3}}{\sqrt{\sum_{b}{\left(Z_{b,i}^{2}\right)}^{2}}\sqrt{\sum_{b}{\left(Z_{b,j}^{3}\right)}^{2}}}\right)}^{2},$$

where *i* and *j* denote the indices of the network output vector dimensions, *b* represents the index of samples within the current batch, and α is a constant used to balance the importance between the first and second terms of the loss function. Here, $\alpha_{1}=\alpha_{2}=\alpha_{3}=0.0002$. $L_{1,2}$, $L_{1,3}$, and $L_{2,3}$ represent the loss values computed from the different pairs of branches. Specifically, $L_{1,2}$ indicates the loss value computed from the first and second branches, $L_{1,3}$ denotes the loss value from the first and third branches, and $L_{2,3}$ represents the loss value from the second and third branches.

Then, the three loss values are weighted and averaged:

(8) $$L_{Tri-ReD}=\frac{1}{N}\sum_{m=1}^{N-1}\sum_{n=m+1}^{N}L_{m,n},$$

where $L_{Tri-ReD}$ represents the weighted average of all loss terms, *N* denotes the total number of branches (N=3 in this paper), and *m* and *n* denote the output indices of the branches. $L_{m,n}$ signifies the loss between the feature *m* and feature *n*, with m≠n.

Specifically, for each set of feature representations obtained, we map them pairwise to derive more targeted losses. These loss values are then accumulated to compute the overall loss, which is averaged by Equation (8).

Different aspects of the correlation among features can be captured by different mapping values. The richer the final feature representations are, the better the model’s ability to distinguish between different categories is. In other words, this loss function is designed not only to consider the information from individual feature vectors but also to more accurately analyze and learn the relationships between different features from multiple perspectives. Consequently, the interactions between features can be better understood by the model.

### 3.3. Experimental Setup

All experiments were conducted under the same hardware and software conditions as follows. GPU: GeForce RTX 3090; OS: Ubuntu 18.04.5 LTS; CUDA Version: 11.4; PyTorch Version: 1.11.0 for cu113; TorchVision Version: 0.12.0 for cu113.

In our study, to maintain consistency with the referenced datasets, the labeled datasets used in downstream tasks were configured as is shown in Table 1. Additionally, all SSL models were pre-trained for 800 epochs. We used the SGD optimizer to fine-tune downstream classification tasks. The batch size for both pre-training and fine-tuning was 128. To ensure a fair comparative analysis of experimental outcomes, results were averaged over ten trials to mitigate the impact of randomness.

To facilitate the evaluation of our proposed method for SVI classification and urban functional zone identification, we selected ResNet18 as the baseline model. ResNet18 has been extensively validated and demonstrates outstanding performance in image classification tasks, making it a promising starting point for comparison. The performance of the self-supervised pre-trained model was evaluated by fine-tuning it in the downstream tasks (street-view image classification and urban functional zone identification) with consistent epochs across experiments. The fine-tuned model was then assessed on the test set of the downstream task. Furthermore, in Phase 2 of Figure 2, the network structure consists of the encoder from Phase 1 and the MLP for final classification. In this paper, two evaluation protocols were employed: the linear and fine-tuning evaluation protocols. For the former one, all layers except the final FC layer were frozen and only the final FC layer was re-trained, while all layers were re-trained in the latter. We employed overall accuracy (OA) as the metric to evaluate the classification performance, along with recall and precision, and calculated the F1-Score. Additionally, we present the duration of the pre-training process, measured in seconds(s).

To further compare and understand the optimal training strategies for the datasets, we conducted experiments by training models from scratch and utilizing the latest versions of pre-trained weights from ImageNet.

## 4. Results

In this section, we describe how a series of comparative and ablation experiments was conducted to verify the effectiveness of Tri-ReD. A detailed analysis of the experimental results is performed, including both quantitative and qualitative assessments.

### 4.1. Comparison Experiment

**Figure 9:**
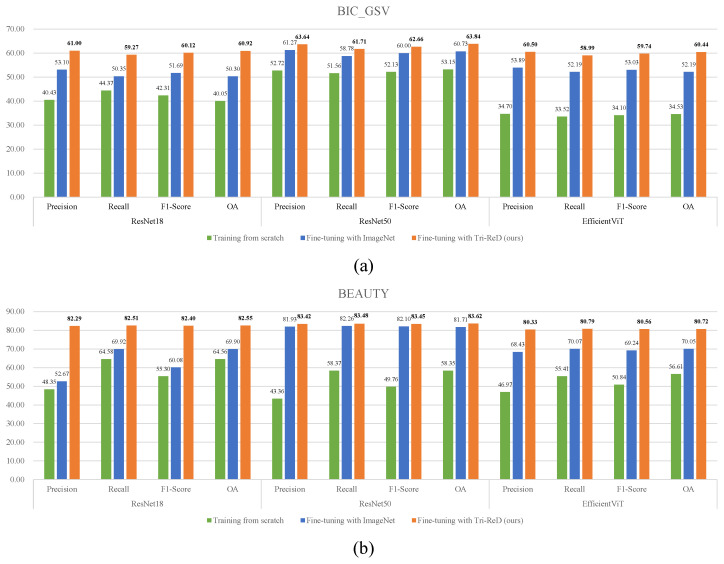
Performance comparison (%) of different backbones on (**a**) BIC_GSV and (**b**) BEAUTY datasets.

From Figure 9, we can observe that the model trained from scratch performed notably poorly on both the urban ground object classification task (BIC_GSV) and functional zone identification task (BEAUTY). Models fine-tuned on ImageNet clearly improved, while our model, pre-trained with SSL, achieved the best results. Apart from this, for the more abstract functional zones like those in the BEAUTY dataset, whether or not the model was trained from scratch or with ImageNet pre-trained weights, the gaps in OA and recall values were relatively small. However, the gaps between OA and precision and the F1-Score are larger. This phenomenon was particularly obvious with the ResNet18 model. When utilizing the ResNet50 model pre-trained with ImageNet weights, there was an improvement.

A reasonable explanation for this phenomenon is that OA reflects the overall classification accuracy of a model across all classes, while precision and recall focus on the classification accuracy of negative and positive instances, respectively. As shown in Figure 7, the problem of class imbalance existed in the BEAUTY dataset. The feature representations extracted from the shallow ResNet18 model were insufficient. Despite the similar values of OA and recall, more false positives were generated by the model during positive instance predictions. This resulted in lower precision values. The overall results of the ResNet50 model were better due to its superior feature extraction capabilities. Although the results were more consistent when using the strategy of fine-tuning with ImageNet, there was still a gap between OA and precision when using the training-from-scratch strategy. The performance of Tri-ReD in EfficientViT outperformed both training from scratch and fine-tuning with ImageNet-pre-trained weights. On the BIC_GSV dataset, OA improved by 25.91% compared to training from scratch and 8.25% compared to ImageNet pre-training. On the BEAUTY dataset, OA rose by 24.11% and 10.67%, respectively. However, its overall performance was slightly lower than ResNet50, which may be attributed to the transformer architecture. Although transformer models excel with large-scale data, they may not fully take advantage of their advantages in smaller datasets or tasks with more localized features.

By employing tri-branch mutually exclusive augmentation and Trilateral Redundancy Reduction loss, more discriminative and representative feature representations could be generated by Tri-ReD. The experimental results show that the values of OA, precision, recall, and F1-Score in Tri-ReD were more consistent and better, whether we used the shallow ResNet18, deeper ResNet50, or transformer-based EfficientViT model.

A comparison between the Tri-ReD method and prevailing SSL methods on the ResNet18 model is shown in Table 2. In the majority of cases, Tri-ReD outperformed others. Regarding ground object classification in the BIC_GSV dataset, compared to MoCo and SimCLR, the performance of Tri-ReD showed a pronounced improvement, with OA increasing by 3.10% and 4.38%, respectively. In comparison to BYOL and Barlow Twins, Tri-ReD exhibited enhancements in OA by 1.07% and 1.78%, respectively. When the dataset for downstream tasks involved more abstract functional zones like the BEAUTY dataset, Tri-ReD also demonstrated its superior performance, improving OA by 6.65% and 12.52% compared to MoCo and SimCLR, respectively. In comparison to BYOL and Barlow Twins, Tri-ReD also showed an improvement of 4.66% and 0.77%, respectively. The above experiments show that our method was applicable to both levels of urban scene classification tasks and performed better for more abstract functional zones.

In this study, in order to explore how different models and different depths or widths of neural networks affected the performance, we conducted experiments on both ResNet50 (the results are shown in Table 3) and EfficientViT [47] (see Table 4) to validate the effectiveness of our proposed method.

From Table 3, we observe that MoCo, SimCLR, and BYOL performed worse in ResNet50 compared to ResNet18. Our explanations for this observation are as follows: Firstly, due to memory limitations, we used a relatively small batch size, only 128. Secondly, the pre-training data we used were also relatively limited, with only about 120,000 images. This was inadequate for larger models like ResNet50, leading to insufficient feature learning and hence poorer performance. Lastly, we did not employ many data augmentation techniques, which may have resulted in some views containing redundant information. The model’s ability to learn differences between views was impaired, which further resulted in the larger model exhibiting unsatisfactory performance in capturing more diverse features.

Despite these constraints, Tri-ReD, a simple redundancy reduction strategy, still achieved optimal results without extremely large pre-training datasets, larger batch sizes, or more extensive data augmentation techniques. As demonstrated by the experimental results in Table 4, Tri-ReD remained applicable and achieved favorable performance even when the backbone model is replaced with EfficientViT. This further substantiates that our framework is architecture-agnostic.

**Figure 10:**
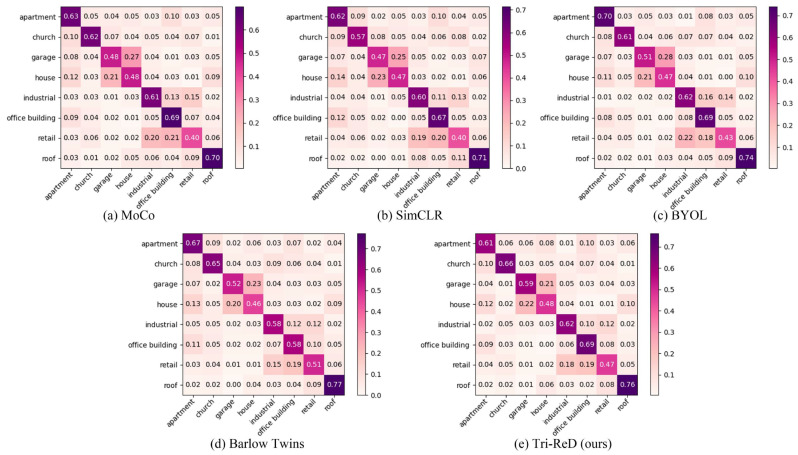
The normalized confusion matrix of the SSL methods (**a**–**e**).

**Figure 11:**
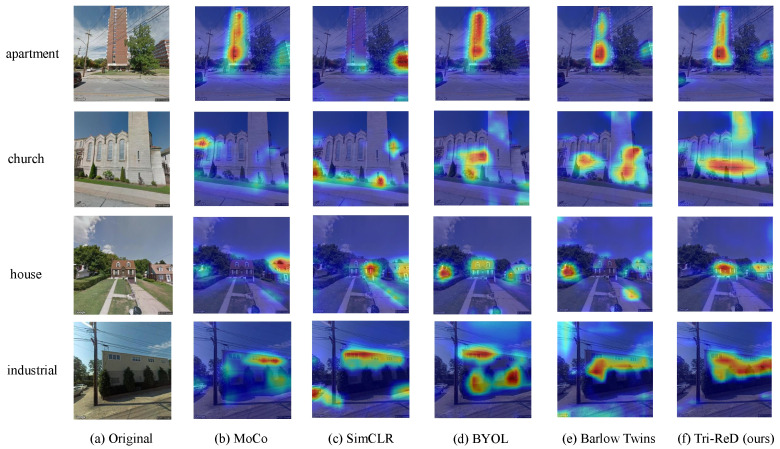
The class activation heatmap of the BIC_GSV dataset from the ResNet18 model.

Figure 10 and Figure 11 present the visualization results of other SSL methods and the Tri-ReD method using the ResNet18 model. The confusion matrix of different SSL methods on the BIC_GSV dataset is shown in Figure 10, while the class activation heatmap for partial samples based on Grad-CAM [48] is shown in Figure 11. It is evident that Tri-ReD outperformed other SSL methods in five to seven categories by observing the confusion matrix in Figure 10. Combined with the heatmaps in Figure 11, we can see that Tri-ReD focused more accurately on the relevant regions. These results highlight the exceptional capabilities of Tri-ReD.

### 4.2. Ablation Experiment

To validate the effectiveness of the Tri-ReD framework and the SVCO method, we conducted ablation experiments using ResNet18. To begin with, we conducted both bi-branch and tri-branch ablations with identical data augmentations; the results are shown in Table 5. Bi-MExA refers to the bi-branch mutually exclusive augmentation. The experimental results from the linear evaluation show that Tri-MExA outperformed previous dual-path SSL approaches, with a 3.95% improvement in OA on the BIC_GSV dataset at the ground object level. For the higher-level functional zones, there was a 1.05% improvement. Subsequently, we assessed the effectiveness of SVCO data augmentation within the tri-branch framework, as shown in Table 6. Applying the SVCO data augmentation led to a 0.84% increase in performance on the BIC_GSV dataset. Additionally, CutMix [49] and MixUp [50], two widely used and established data augmentation methods, were chosen for comparison. In each case, SVCO in the third branch was replaced with one of these methods. The results on the BIC_GSV dataset show that SVCO outperformed CutMix by 0.5% in OA and outperformed MixUp by 0.81%. On the BEAUY dataset, SVCO outperformed CutMix by 0.54% and MixUp by 1.1%. CutMix and MixUp were selected due to their proven effectiveness in enhancing model generalization. The comparison highlights the advantages of SVCO, particularly in improving model robustness. It is worth mentioning that, although the occlusion phenomenon in the BEAUTY dataset was less pronounced compared to BIC_GSV, a 0.57% improvement was observed in urban functional zone identification.

The experiments indicated that our Tri-ReD achieved outstanding performance, rendering it an effective and efficient SSL method.

## 5. Discussion

**Figure 12:**
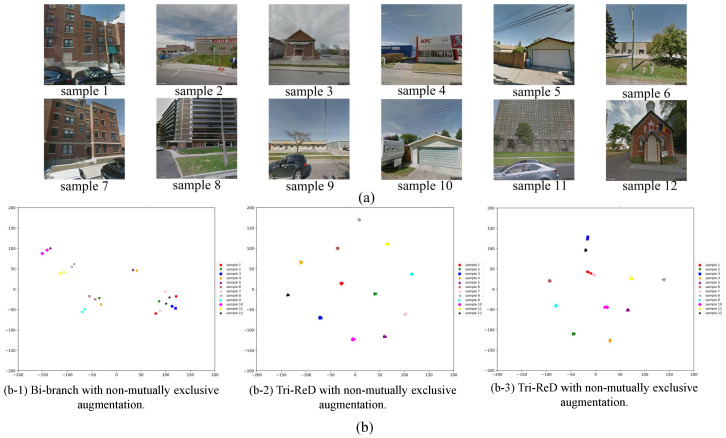
t-SNE visualization of feature distributions for 12 samples from CSM dataset: (**a**) sample presentation; (**b**) t-SNE visualization of feature distributions; (**b-1**) bi-branch strategy with non-mutually exclusive data augmentation; (**b-2**) Tri-ReD strategy with non-mutually exclusive data augmentation; and (**b-3**) Tri-ReD strategy with mutually exclusive data augmentation.

Samples are shown in Figure 12a. In Figure 12b, identical colors and shapes represent different augmented views of the same sample, while distinct markers indicate different samples. The different augmented views of the same sample (shown in Figure 12(b-1) for two views and Figure 12(b-2,b-3) for three views) should ideally be closer to each other in the sample space.

In Figure 12(b-1), the clustering primarily relies on visual similarity. For instance, a group of samples (samples 2, 4, 6, and 9) in the lower-left corner is clustered based on the shape of buildings. However, a closer inspection reveals that samples 2 and 4 clearly correspond to “retail”, while samples 6 and 9 represent “industrial”. Additionally, the cluster on the right (samples 1, 2, 3, 7, and 12) shows suboptimal separation. Particularly, samples 2 and 12, despite exhibiting considerable visual differences, fail to be effectively distinguished. In summary, although some distinctions are made visually, the overall separability is limited and the results remain unsatisfactory. When implementing the more restrictive Tri-ReD strategy, the distance between samples from the same view is further diminished, leading to fantastic clustering outcomes (refer to Figure 12(b-2)). However, although samples 2 and 9 are more clearly separated, this approach tends to treat each sample as an individual class, which leads to excessive separation between samples of different categories. In other words, visually similar samples are not grouped together, resulting in an overall clustering performance that remains suboptimal. When combined with the mutually exclusive data augmentation strategy, the features of similar samples are clustered together. Figure 12(b-3) demonstrates a significant improvement. For example, samples 1 and 7 in Figure 12a exhibit stronger visual consistency, which suggests that their different views should be closer together in the sample space. Furthermore, they show significant visual differences from other samples, resulting in greater distinctiveness in the feature space. The result in Figure 12(b-3) clearly outperforms those in Figure 12(b-1,b-2). This shows that meaningful visual features were effectively extracted by the pre-trained model using the proposed self-supervised method. In conclusion, the more restrictive redundancy reduction loss function of Tri-ReD yielded near-perfect clustering results. The model learned more compact and effective representations with stronger restriction, simplifying the redundancy reduction process. Additionally, the mutually exclusive data augmentation strategy forced the model to learn complementary features across different views, further reducing feature redundancy. This strategy not only allowed the model to capture finer-grained distinctions between similar class samples but also enhanced its performance in classification tasks, allowing for more accurate differentiation between classes.

The t-SNE visualizations demonstrate the effectiveness of Tri-ReD and further validate the design concept presented in Section 3.2.2 and Section 3.2.3. In Section 4, we validate the effectiveness of Tri-ReD through experimental results. To further investigate how Tri-ReD enhanced restriction and simplifies redundancy reduction, we employed t-SNE to visualize the feature distribution outputs, as illustrated in Figure 12. These features were extracted by the SSL pre-training backbone. Figure 12a shows 12 samples from the CSM dataset, while Figure 12b presents the t-SNE visualization results of these 12 samples.

## 6. Conclusions

AI technology is improving in leaps and bounds, and we now have easy access to vast amounts of data such as street-view images, which are increasingly utilized for various remote sensing tasks like scene classification. However, labeling these data is notably time-consuming and labor-intensive. Moreover, issues related to data quality (e.g., severe occlusions) have become more prominent. Consequently, it has become a popular research focus to use SSL methods that do not require labeled information to improve street-scene image classification performance. In this paper, we introduce SSL to fully utilize large volumes of unlabeled data for model pre-training. Specifically, Tri-ReD, a method that eliminates the need for negative samples and complex tricks, is proposed in this work. Due to restrictive Trilateral Redundancy Reduction loss, positive feature representations are pulled closer together in the feature space. Additionally, we introduce SVCO, which is specifically designed for generating samples that better mimic real-world scenarios with severe occlusions. This not only improves the model’s robustness to occlusion but also enhances its generalization ability in diverse scenes. Thanks to these innovations, Tri-ReD achieved the best performance on both datasets.

In summary, SSL remains a vibrant and promising field of research. There is potential for numerous innovative methods and applications to emerge in the future. In subsequent work, we aspire to combine geolocation data to further refine the delineation of urban functional zones.

## Figures and Tables

**Table 1:** The labeled dataset after splitting.

Datasets	Total	Training		Testing
		Training	Validation	Testing
BIC_GSV	19,658	17,600	-	2058
BEAUTY	19,070	12,871	1431	4768

**Table 2:** Classification performance on the BIC_GSV and BEAUTY datasets under the linear and fine-tuning evaluation protocols in the ResNet18 model.

Pre-Training Method	Pre-Training Time (s)	BIC_GSV				BEAUTY			
		OA		Kappa		OA		Kappa	
		Linea	Fine-Tuning	Linear	Fine-Tuning	Linear	Fine-Tuning	Linear	Fine-Tuning
MoCo	386,666	28.33%	57.82%	17.76%	48.77%	65.33%	75.90%	41.79%	63.17%
SimCLR	243,866	28.28%	56.54%	18.03%	47.27%	52.29%	70.03%	14.63%	52.28%
BYOL	236,460	26.82%	59.85%	15.28%	52.19%	57.99%	77.89%	26.59%	66.57%
Barlow Twins	557,177	48.20%	59.14%	40.88%	51.70%	68.04%	81.78%	48.81%	72.88%
**Tri-ReD** (ours)	247,731	49.63%	60.92%	42.05%	53.41%	68.90%	82.55%	49.26%	73.86%

**Table 3:** Classification performance on the BIC_GSV and BEAUTY datasets under the linear and fine-tuning evaluation protocols in the ResNet50 model.

Pre-Training Method	Pre-Training Time (s)	BIC_GSV				BEAUTY			
		OA		Kappa		OA		Kappa	
		Linea	Fine-Tuning	Linear	Fine-Tuning	Linear	Fine-Tuning	Linear	Fine-Tuning
MoCo	433,170	25.02%	54.89%	13.38%	45.28%	59.92%	66.95%	30.36%	45.64%
SimCLR	612,658	26.77%	51.03%	15.78%	41.58%	45.60%	66.49%	0.17%	45.04%
BYOL	811,727	23.81%	53.12%	13.74%	43.07%	49.92%	66.21%	9.41%	44.40%
Barlow Twins	688,922	52.43%	62.19%	45.62%	54.43%	78.19%	82.71%	66.66%	74.81%
**Tri-ReD** (ours)	779,775	54.03%	63.84%	47.46%	56.22%	79.03%	83.62%	68.00%	75.96%

**Table 4:** Classification performance on the BIC_GSV and BEAUTY datasets under the linear and fine-tuning evaluation protocols in the EfficientViT model.

Pre-Training Method	Pre-Training Time (s)	BIC_GSV				BEAUTY			
		OA		Kappa		OA		Kappa	
		Linea	Fine-Tuning	Linear	Fine-Tuning	Linear	Fine-Tuning	Linear	Fine-Tuning
MoCo	233,670	40.82%	51.45%	32.50%	42.01%	66.97%	70.53%	45.65%	54.37%
SimCLR	232,210	32.22%	48.82%	22.67%	38.47%	65.14%	68.14%	42.41%	49.62%
BYOL	500,143	29.79%	56.77%	19.88%	48.15%	63.68%	77.92%	39.92%	66.48%
Barlow Twins	284,428	44.66%	59.25%	36.81%	50.94%	72.47%	80.00%	58.74%	70.01%
**Tri-ReD** (ours)	242,268	46.02%	60.44%	38.38%	51.99%	73.30%	80.72%	59.83%	70.87%

**Table 5:** Ablation of Trilateral Redundancy Reduction framework under linear and fine-tuning evaluation protocols.

Num_Branches	BIC_GSV				BEAUTY			
	OA		Kappa		OA		Kappa	
	Linear	Fine-Tuning	Linear	Fine-Tuning	Linear	Fine-Tuning	Linear	Fine-Tuning
Bi-MExA	45.68%	59.25%	38.65%	51.47%	67.85%	81.69%	48.01%	72.82%
Tri-MExA	49.63%	60.92%	42.05%	53.41%	68.90%	82.55%	49.26%	73.86%

**Table 6:** Ablation of simulating vegetation color occlusion under linear and fine-tuning evaluation protocols. (The ✓ indicates the inclusion of the listed data augmentation).

Augmentation				BIC_GSV				BEAUTY			
Basic Augmentation Resize and Crop	Branch 1 Horizontal Flip	Branch 2 Color Jitter	Branch 3 Aug.	OA		Kappa		OA		Kappa	
				Linear	Fine-Tuning	Linear	Fine-Tuning	Linear	Fine-Tuning	Linear	Fine-Tuning
✓	✓	✓	**-**	48.74%	60.08%	41.09%	52.39%	68.54%	81.98%	48.61%	73.12%
✓	✓	✓	CutMix	49.01%	60.42%	41.49%	52.68%	68.69%	82.01%	48.89%	73.46%
✓	✓	✓	MixUp	48.82%	60.11%	41.13%	52.35%	68.52%	81.45%	48.56%	72.99%
✓	✓	✓	SVCO	49.63%	60.92%	42.05%	53.41%	68.90%	82.55%	49.26%	73.86%

## Data Availability

Some useful information is also available at https://github.com/yira-Lee/Tri-ReD (accessed on 25 February 2025).
